# Cytosolic group IVa phospholipase A_2 _mediates IL-8/CXCL8-induced transmigration of human polymorphonuclear leukocytes *in vitro*

**DOI:** 10.1186/1476-9255-7-14

**Published:** 2010-03-18

**Authors:** Angelo Y Meliton, Nilda M Muñoz, Lucille N Meliton, David C Binder, Christopher M Osan, Xiangdong Zhu, Steven M Dudek, Alan R Leff

**Affiliations:** 1 Section of Pulmonary and Critical Care Medicine, Departments of Medicine, The University of Chicago, 5841 S Maryland Avenue, MC 6026, Chicago, IL 60637, USA; 2 Pediatrics, Pharmacology and Physiology and Committees on Molecular Medicine, Clinical Pharmacology and Pharmacogenomics, and Cell Physiology, The University of Chicago, 5841 S Maryland Avenue, MC 6026, Chicago, IL 60637, USA

## Abstract

**Background:**

Cytosolic gIVaPLA_2 _is a critical enzyme in the generation of arachidonate metabolites and in induction of β_2_-integrin adhesion in granulocytes. We hypothesized that gIVaPLA_2 _activation also is an essential downstream step for *post adhesive *migration of PMN *in vitro*.

**Methods:**

Migration of PMNs caused by IL-8/CXCL8 was assessed using a transwell migration chamber. PMNs were pretreated with two structurally unrelated inhibitors of gIVaPLA_2_, arachidonyl trifluoromethylketone (TFMK) or pyrrophenone, prior to IL-8/CXCL8 exposure. The fraction of migrated PMNs present in the lower chamber was measured as total myeloperoxidase content. GIVaPLA_2 _enzyme activity was analyzed using [^14^C-PAPC] as specific substrate F-actin polymerization and cell structure were examined after rhodamine-phalloidin staining.

**Results:**

IL-8/CXCL8-induced migration of PMNs was elicited in concentration- and time-dependent manner. Time-related phosphorylation and translocation of cytosolic gIVaPLA_2 _to the nucleus was observed for PMNs stimulated with IL-8/CXCL8 in concentration sufficient to cause upstream phosphorylation of MAPKs (ERK-1/2 and p38) and Akt/PKB. Inhibition of gIVaPLA_2 _corresponded to the magnitude of blockade of PMN migration. Neither AA nor LTB_4 _secretion was elicited following IL-8/CXCL8 activation. In unstimulated PMNs, F-actin was located diffusely in the cytosol; however, a clear polarized morphology with F-actin-rich ruffles around the edges of the cell was observed after activation with IL-8/CXCL8. Inhibition of gIVaPLA_2 _blocked change in cell shape and migration caused by IL-8/CXCL8 but did not cause F-actin polymerization or translocation of cytosolic F-actin to inner leaflet of the PMN membrane.

**Conclusion:**

We demonstrate that IL-8/CXCL8 causes a) phosphorylation and translocation of cytosolic gIVaPLA_2 _to the nucleus, b) change in cell shape, c) polymerization of F-actin, and d) chemoattractant/migration of PMN in vitro. Inhibition of gIVaPLA_2 _blocks the deformability and subsequent migration of PMNs caused by IL-8/CXCL8. Our data suggest that activation of gIVaPLA_2 _is an essential step in PMN migration *in vitro*.

## Background

IL-8/CXCL8 is a selective and potent neutrophil chemoattractant. Previous studies have shown that upstream activation of PI3K, ERK-1/2, or p38 MAPK [1-7] pathways caused by IL-8/CXCL8 regulates the induction of transendothelial PMN migration. However, the signaling mechanism downstream of these kinases in causing migration of PMNs has not been established previously and critical intermediate steps regulating neutrophil migration remain unknown.

Phospholipase A_2_s (PLA_2_) are esterases that cleave glycerophospholipids at the *sn*-2 ester bond, releasing a fatty acid and a lysophospholipid [8-11]. PLA_2_s are divided into five different groups; a) secretory PLA_2 _[12,13], b) cytosolic gIVPLA_2 _(gIVPLA_2_) [14], c) Ca^2+^-independent PLA_2_, [15,16] d) platelet-activating factor hydrolyses, [17,18] and e) lysosomal PLA_2 _[19]. Among these groups, gIVaPLA_2 _is thought to be not only a rate-limiting enzyme in eicosanoid biosynthesis [20] but also to be essential in maintenance of β_2_-integrin adhesion in granulocytes [21,22]. We have shown previously that ERK-1/2 and Akt/PKB phosphorylation activated gIVaPLA_2 _to cause β_2_-integrin adhesion of granulocytes to ICAM-1[23]. We also have shown that phosphorylation to activate gIVaPLA_2 _results from upstream phosphorylation of these kinase and that maintenance of this phosphorylated state regulates the process of β_2_-integrin adhesion [24,25].

Because MAP kinase and PI3K also regulate gIVaPLA_2 _phosphorylation, we postulated that activation of gIVaPLA_2 _might regulate neutrophil migration. The objective of this study was to examine specifically the functional role of gIVaPLA_2 _in PMN *migration *caused by IL-8/CXCL8. IL-8/CXCL8 was applied in concentration causing upstream phosphorylation of ERK-1/2, p38 MAPK and Akt/PKB. We found that inhibition of gIVaPLA_2 _activity blocked substantially the transmigration toward IL-8/CXCL8 in a transwell chamber. This study is the first demonstration that activation of gIVaPLA_2 _is a critical regulatory step subsequent to upstream activation of signaling kinases in eliciting PMN migration.

## Methods

### Antibodies and reagents

IL-8/CXCL8 was purchased from Peprotech (Rocky Hill, NJ) while bovine serum albumin fraction V and human polymophonuclear leukocytes (PMNs) isolation materials were purchased from Sigma-Aldrich Chemical Co. (St. Louis, MO). Anti-phosphorylated gIVaPLA_2 _Ab (Ser^505^) was purchased from Cell Signaling Technology (Beverly, MA). Mouse IgG was purchased from BD Biosciences (Mountain View, CA). Polystyrene 96-well microtiter plates were obtained from Neuro Probe (Gaithersburg, MD). Rhodamine-phalloidin was obtained from Sigma-Aldrich Chemical (St. Louis, MO).

### Isolation of human PMNs

Venous blood from normal human subjects (20-45 years old) was collected in heparin-containing tubes, and PMNs were isolated by Ficoll-Paque sedimentation as described previously [26,27]. Purity of PMN on H and E-stained cytoslides was ~90-95%. Informed written consent was obtained from all volunteers in this study.

### Transwell migration assay

PMN migration in transwell microplates was assessed using the standard methods as described previously [28]. Preliminary experiments have established that the number of cells (4 × 10^4 ^cells) used allow the optimal % cell migration without clogging the pores of transwell filter of the upper chamber. Cells then were preincubated with HBSS, 3 μM - 30 μM arachidonyl trifluoromethylketone [TFMK; inhibitor of gIVaPLA_2 _[29], or 10^-9 ^M -10^-6 ^M pyrrophenone [inhibitor of gIVaPLA_2 _[30] for 30 min at 37°C. Treated cells in 50 μl HBSS were transferred onto 5 μm-pore transwell filters positioned on top of the migration chamber. HBSS or 10 ng/ml to 1000 ng/ml IL-8/CXCL8 was loaded in the bottom chamber (final volume = 310 μl), and the transwell microplates were incubated for 60 min and 90 min at 37°C. The migrated PMNs were treated with 100 μl of HBSS + 10% FBS buffer and 100 μl developing solution [8 ml 100 nM NaH_2_PO_4 _(pH = 5.5), 1000 μl 10% hexadecyltrimethylammonium bromide (HTAB), 3 μl 30% hydrogen peroxide, 1000 μl 10% *o*-dianisidine dihydrochloride]. The reaction was terminated by addition of 50 μl sulfuric acid and myeloperoxidase (MPO) activity was measured at 405 nm in a Thermomax microplate reader (Molecular Devices, Menlo Park, CA). The fraction of migrated PMNs present in the lower chamber was measured as total MPO content. Data were expressed as % cell migration. Maximal, no-toxic inhibitory concentration of TFMK and pyrrophenone were established in initial studies demonstrating blockade of gIVaPLA_2 _activity [see also Results].

In separate studies, morphological changes of the non-migrated cells (top chamber) and migrated cells (bottom chamber) toward IL-8/CXCL8 were examined. The effect of 30 μM TFMK or 10^-6 ^M pyrrophenone on cell deformability caused by IL-8/CXCL8 also was examined using confocal microscopy.

### Immunoblotting analysis

PMNs (10^6 ^cells/group) were activated with HBSS and 100 ng/ml IL-8/CXCL8 at different time intervals, and phosphorylation of ERK-1/2, p38 MAPK, and Akt/PKB were analyzed using Western blot. The pellet was lysed in disruption buffer (20 mM Tris-HCl, 30 mM Na_4_P_2_O_7_, 50 mM NaF, 40 mM NaCl, 5 mM EDTA, pH 7.4) containing 1% Nonidet P-40, 10 μg/ml leupeptin, 5 μg/ml aprotinin, 1 mM PMSF, 2 mM Na_3_VO_4_, and 0.5% deoxycholic acid. Samples were loaded to SDS-PAGE using 8% (gIVaPLA_2_) or 10% (ERK-1/2, p38 MAPK, PI3K) acrylamide gels under reducing conditions. The membrane was blocked with 1% BSA in TBS-T buffer and phosphorylated Ab against ERK-1/2 (1:1000; Cell Signaling Technology; Beverly, MA), p38 MAPK and Akt/PKB (1:1000; Cell Signaling Technology; Beverly, MA), or Ser^505 ^gIVaPLA_2 _(1:1000) was added followed by relevant secondary Ab conjugated with HRP. Protein of interest was analyzed by an enhanced chemiluminescence system (Amersham, Arlington Heights, IL).

### Measurement of arachidonic acid (AA) release

Isolated PMNs were incubated in RPMI medium containing 5% FBS and 0.5 μCi [^3^H]AA. Labeled PMNs were incubated for 2 h and unincorporated [^3^H]AA was washed away by HBSS containing 0.2% BSA. Thereafter, treated PMNs were activated with saline, 100 ng/ml IL-8/CXCL8 or 1 μM FMLP (+ 5 μg/ml cytochalasin B). The reaction was terminated by centrifugation at 12,000 × *g *for 1 min. Supernatant were collected, and pellets were lysed in 1% Triton X-100. [^3^H]AA release was measured by scintillation counting and expressed as counts per min (cpm) [21,24].

### Measurement of LTB_4 _secretion

Aliquots of 250,000 were activated with saline, 1-1000 ng/ml IL-8/CXCL8, or 1 μM FMLP (+ 5 μg/ml cytochalasin B) for 15 min at 37°C in a final volume of 250 μl HBSS. The reaction was terminated by centrifugation at 12,000 × *g *for 1 min. Aliquots of supernatants were assayed with a commercial EIA kit as previously described [24,31].

### GIVaPLA_2 _activity assay

GIVaPLA_2 _activity assay was determined in aliquots of 2 × 10^6 ^cells incubated for 15 min at 37°C with 3 μM - 30 μM TFMK or 10^-10 ^M - 10^-6 ^M pyrrophenone. Activity was measured at optimal time (30 min) after 100 ng/ml IL-8/CXCL8 [see Results]. This time and concentration were shown in initial studies to cause phosphorylation of gIVaPLA_2 _in PMNs. The cell pellets were resuspended in disruption buffer (see above) and immediately sonicated followed by addition of specific substrate ([^14^C]-PAPC) for gIVaPLA_2_[24,31]. To measure precisely the total gIVaPLA_2 _activity, 5 mM dithiotrietol was added to cell lysate to inactivate, if any, the remaining 10-14 kDa secretory PLA_2 _enzymes that could interfere with the assay. Thirty min later, the reaction was terminated by adding 560 μl of Dole's reagent (heptane:isopropyl alcohol:1 N H_2_SO_4_; 400:390:10 by vol), and the radioactivity was measured in a liquid scintillation counter and expressed as picomoles/30 min/10^6 ^cells [31].

### Subfractionation

Freshly isolated PMNs were preincubated with either saline, 100 ng/ml IL-8/CXCL8, or 10^-6 ^M FMLP for 15 min at 37°C. After washing with PBS, treated cells were centrifuged for 1 min at 400 × *g*. The pellet were lysed in 50 μl disruption buffer (see above) and put on ice for 10 min. The disrupted pellets, which are mainly nuclear component of the cells, were centrifuged at 500 × *g *for 1 min. A total of 50 μl of boiling buffer was added to the pellets and boiled for 5 min. The supernatants were centrifuged again at 100,000 × *g *for 1 h. Eight μl of loading buffer was added to the collected supernatant, which is the cytoplasm fraction, and was boiled for 5 min. Samples were loaded onto SDS-PAGE and membrane was probed with pAb against cPLA_2_. The translocation of cytosolic gIVaPLA_2 _to the nuclear component of the cells was detected by an enhanced chemiluminescence (Amersham, Arlington Heights, IL).

### Change in cell shape and F-actin polymerization

Change in cell shape and F-actin polymerization were examined in migrated cells. HBSS or rhodamine-phalloidin [32] was added to the paraformadehyde-fixed cytoslides containing samples and changes in cell shape and F-actin polymerization were analyzed by confocal microscopy.

### Statistical analysis

Experimental data are expressed as mean ± SEM in each group. Student's *t-test *was used for comparison between two-paired groups. Where multiple comparisons were made, differences on concentration-response curves for the same agonist or inhibitor were compared after Bonferonni correction. Variation between more than two groups was tested using one-way ANOVA followed by Fisher' *least *protected difference test. Statistical significance was claimed when P < 0.05.

## Results

### IL-8/CXCL8-induced migration

**Figure 1:**
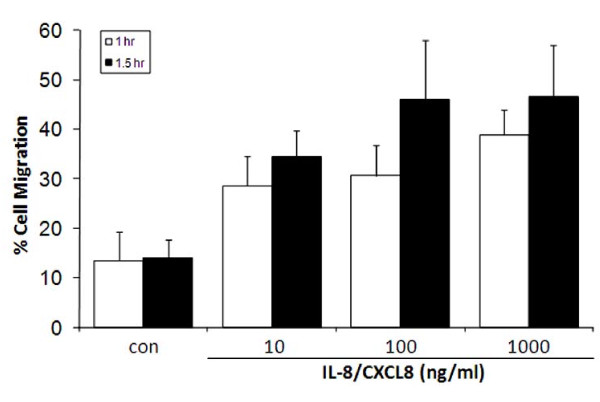
**IL-8/CXCL8-induced neutrophil (PMN) migration**. The concentration-response curve to IL-8/CXCL8-induced PMN migration was generated at 60 min or 90 min, and migration was assessed by measuring total myeloperoxidase activity in the neutrophil fraction (see Methods section). Data are expressed as % cell migration from N = 5 isolations.

Migration of PMNs toward the IL-8/CXCL8 chamber increased in time- and concentration-dependent manner (Figure 1). Transmigration caused by 100 ng/ml IL-8/CXCL8 was substantially greater after 90 min incubation time compared to 60 min incubation time. PMN migration across the transwell filter in the absence of IL-8/CXCL8 was < 15%. Transmigrated PMNs increased from 14.0 ± 3.7% to 46.0 ± 11.9% after 100 ng/ml IL-8/CXCL8 (P < 0.05); 1000 ng/ml IL-8/CXCL8 had no further effect on migration on PMNs. Accordingly, a 90 min incubation time and a concentration of 100 ng/ml IL-8/CXCL8 were used for all subsequent migration assays.

### IL-8/CXCL8-induced upstream kinases phosphorylation

**Figure 2:**
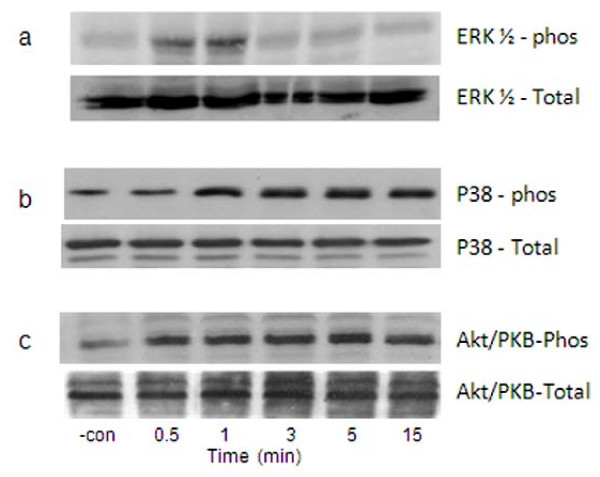
**Phosphorylation (phos) of ERK-1/2, p38 MAPK, and Akt/PKB caused by IL-8/CXCL8**. Whole cells extracts were prepared from isolated PMN treated with 100 ng/ml IL-8/CXCL8 at different times. Lysed PMNs were then stained with specific pAb directed against phosphorylated a) ERK-1/2, b) p38 MAPK or c) Akt/PKB. Equal loading of sample is demonstrated by staining of total protein for each kinase. Results shown are representative immunoblots obtained from 4 healthy donors. Negative control (-con) is buffer-activated cells.

We first demonstrated that the 100 ng/ml concentration of IL-8/CXCL8 used in these studies caused phosphorylation of critical upstream kinases, ERK-1/2, p38 MAPK and Akt/PKB (a target protein for PI3K), in the same PMN isolates (Figure 2). Phosphorylated ERK-1/2 was greatest at 0.5-1 min and gradually decreased thereafter. The p38 MAPK and Akt/PKB were constitutively expressed in unstimulated PMNs. Treatment with IL-8/CXCL8 upregulated the phosphorylation of p38 MAPK and Akt/PKB, which unlike ERK-1/2, was sustained for ≥ 15 min. Total protein for ERK-1/2, p38 MAPK, and Akt/PKB was stained with respective Ab to demonstrate equal loading of samples.

### Effect of IL-8/CXCL8 on [^3^H]AA release and LTB_4 _secretion

Non-stimulated PMNs released minimal amounts of AA and undetectable amounts of LTB_4_. Activation with IL-8/CXCL8 did not elicit secretion of either AA or LTB_4 _in PMNs. All treated PMNs remained viable as assessed by trypan blue exclusion dye analysis. Measurements were performed as described in Methods section.

### IL-8/CXCL8-induced gIVaPLA_2 _phosphorylation

**Figure 3:**
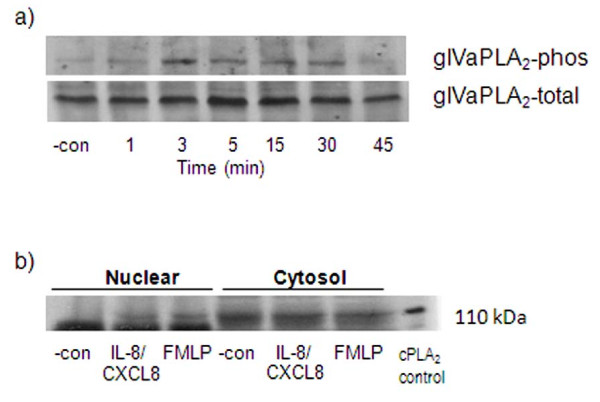
**Phosphorylation (phos) of group IVa phospholipase A_2 _(gIVaPLA_2_) caused by IL-8/CXCL8 activation**. a) Treated PMNs were activated with 100 ng/ml IL-8/CXCL8 at different time intervals, in minutes (min) and were lysed immediately. Treated samples were subjected to Western blot analysis and probed with pAb directed against Ser^505 ^gIVaPLA_2 _(top) or pAb against total gIVaPLA_2 _(bottom). Total gIVaPLA_2 _protein was stained with pAb directed against total gIVaPLA_2 _to indicate equal loading of samples. Representative immunoblot from 3 independent experiments. b) Translocation of cytosolic gIVaPLA_2 _to the nuclear component of PMNs was examined by subfractionation. Representative blot from 3 different donors was shown after staining with mAb against gIVaPLA_2_.

Cytosolic gIVaPLA_2 _is a downstream target of ERK-1/2, p38 MAPK and PI3K during cell adhesion [21,23,25]. We next determined whether gIVaPLA_2 _was a downstream regulator of PMNs *in vitro*. Activation of PMNs with IL-8/CXCL8 caused rapid phosphorylation of gIVaPLA_2 _(Figure 3a). The phosphorylated Ser^505 ^110-kDa-gIVaPLA_2 _protein was greatest at 3 min and was sustained ≥ 30 min; thereafter, the gIVaPLA_2 _phosphorylation decreased to baseline level. Total gIVaPLA_2 _proteins stained with Ab against this protein demonstrated equal loading of samples onto SDS-PAGE.

Immunoblotting analysis of cell-fractional components demonstrated that gIVaPLA_2 _is located mainly in cytoplasm of unstimulated PMNs. Application of IL-8/CXCL8 translocated the cytosolic gIVaPLA_2 _to some extent, to the nuclear component of PMNs (Figure 3b). We used FMLP as a positive control since we previously have shown that FMLP causes the translocation of cytosolic gIVaPLA_2 _to nuclear membrane in eosinophils [33].

### Inhibition of stimulated gIVaPLA_2 _activity

**Figure 4:**
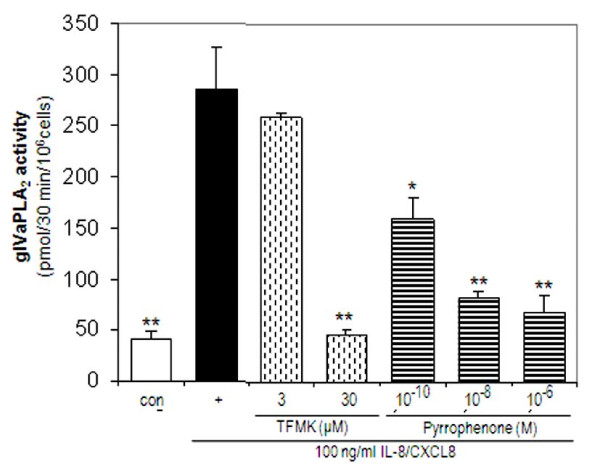
**IL-8/CXCL8-elicited gIVaPLA_2 _enzyme activity**. PMNs were pretreated with either 3 μM - 30 μm arachidonyl trifluoromethylketone (TFMK) or pyrrophenone for 30 min prior to IL-8/CXCL8 activation. Cytosolic gIVaPLA_2 _activity was expressed as picomole (pmol) arachidonic acid (AA)/30 min/10^6 ^cells [31]. Results are the mean ± SEM from N = 5 donor isolations. *P < 0.05 vs IL-8/CXCL8-activated PMNs; **P < 0.01 vs IL-8/CXCL8 -activated PMNs.

In separate studies, the inhibitory effect of TFMK and pyrrophenone on stimulated gIVaPLA_2 _activity in PMNs was determined. Baseline gIVaPLA_2 _activity was 41.0 ± 7.57 pmol/30 min/10^6 ^cells. Activation of PMNs with 100 ng/ml IL-8/CXCL8 increased the gIVaPLA_2 _activity to 288.3 ± 83.6 pmol/30 min/10^6 ^cells (P < 0.001 vs baseline). TFMK (30 μM) caused inhibition to baseline level of gIVaPLA_2 _activity caused by IL-8/CXCL8 (Figure 4; P < 0.01 vs IL-8/CXCL8-activated PMNs; P = NS vs baseline). Pyrrophenone, a potent gIVaPLA_2 _inhibitor, attenuated gIVaPLA_2 _activity in concentration-dependent manner. Pretreatment with 10^-10 ^M pyrrophenone inhibited stimulated gIVaPLA_2 _activity to 160.0 ± 20.1 pmol/30 min/10^6 ^cells (P < 0.05 vs IL-8/CXCL8 alone) and further to 67.5 ± 16.6 pmol/30 min/10^6 ^cells with 10^-6 ^M pyrrophenone (P < 0.01 vs IL-8/CXCL8 alone). All treated PMNs remained viable as assessed by trypan blue exclusion dye analysis.

### Blockade of IL-8/CXCL8-induced PMN migration

**Figure 5:**
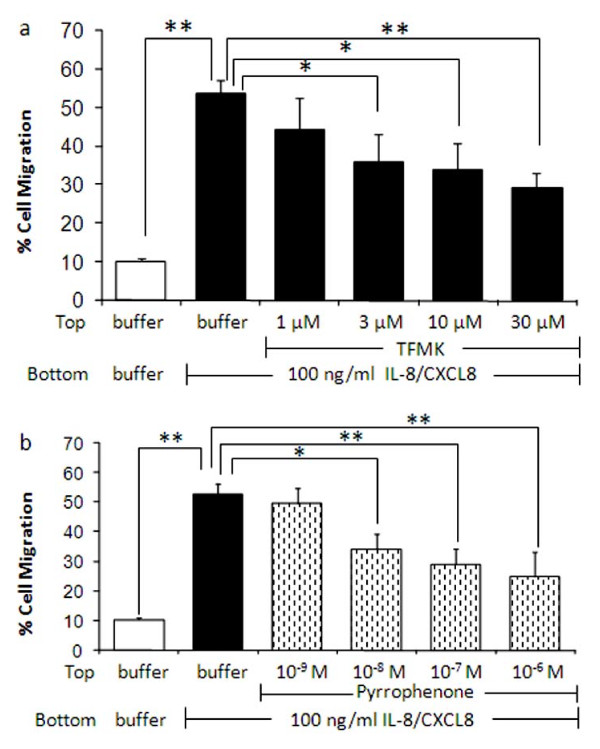
**Blockade of IL-8/CXCL8-induced cell migration by arachidonyl trifluoromethylketone (TFMK) or pyrrophenone**. PMNs were pretreated with 1 μM - 30 μM TFMK or 10^-9 ^M - 10^-6 ^M pyrrophenone for 30 min prior to IL-8/CXCL8 exposure. PMN migration was assessed by measuring the total myeloperoxidase activity in the neutrophil fraction (see Methods section). Data are expressed as % cell migration from N = 6 isolations. *P < 0.05; **P < 0.01 as indicated.

We next determined whether activation of gIVaPLA_2 _is required for *in vitro *migration of PMNs. PMNs first were co-incubated with TFMK or pyrrophenone, and migration to 100 ng/ml IL-8/CXCL8 was analyzed as % of total PMNs in the upper chamber prior to treatment (Figure 5). TFMK (Figure 5a) or pyrrophenone (Figure 5b) attenuated the cell migration toward the IL-8/CXCL8 chamber in concentration-dependent manner. PMN migration caused by IL-8/CXCL8 was 53.6 ± 3.5% compared to 10.3 ± 0.4% for PMNs in a buffer control chamber (P < 0.01). Migration was attenuated to 35.7 ± 7.3% after 3 μM TFMK (P < 0.05 vs IL-8/CXCL8 alone) and further blocked to 29.13 ± 4.15% for cells pretreated with 30 μM TFMK prior to IL-8/CXCL8 exposure (P < 0.01 vs IL-8/CXCL8 alone). Treatment with 10^-8 ^M pyrrophenone blocked the migration to 34.1 ± 3.4% (P < 0.05 vs IL-8/CXCL8 alone) and further to 24.9 ± 8.4% with 10^-6 ^M pyrrophenone (P < 0.01 vs IL-8/CXCL8 alone). These data demonstrate that gIVaPLA_2 _activation is a significant step in neutrophil migration elicited by IL-8/CXCL8 in vitro.

### Cell morphology and F-actin polymerization

**Figure 6:**
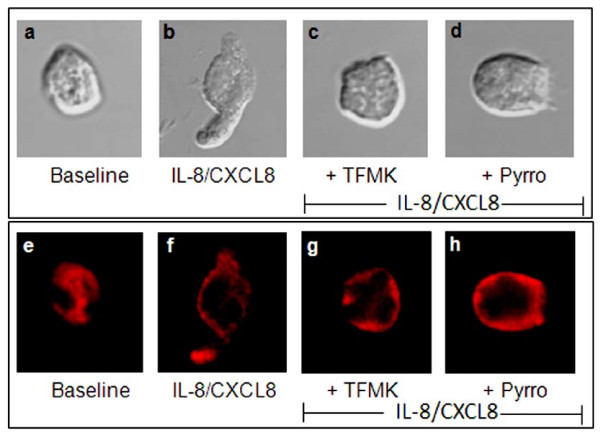
**Change in cell shape and F-actin polymerization of treated PMNs**. (**a-d**) At 90 min incubation time, migrated PMNs (N = 4 donors) were harvested from the IL-8/CXCL8 chamber of the transwell migration apparatus. Change in cell shape was examined by confocal microscopy. Resting PMNs treated with either buffer (a; baseline), 30 μM TFMK (c) or (d) 10^-6 ^M pyrrophenone (pyrro) for 30 min prior to migration toward IL-8/CXCL8. Treated PMNs were then fixed with 10% paraformaldehyde solution and cytoslides were prepared to examine the change in cell shape. For all groups, baseline is buffer-treated cells. Photomicrographs depict high power (630× magnification). (**e-h**) Representative confocal photomicrographs of F-actin polymerization caused by buffer (**e**) or IL-8/CXCL8 (**f**) in the presence or absence of arachidonyl trifluoromethylketone (TFMK; **g**) or pyrrophenone (pyrro; **h**). For all groups, baseline is buffer-treated cells (**e**). Photomicrographs depict high power (630× magnification).

We next examined the change in cell shape of migrated PMNs co-incubated with HBSS, TFMK, or pyrrophenone prior to buffer control or IL-8/CXCL8 exposure at 90 min. Representative photomicrographs of cell morphology are shown in Figure 6. PMNs in the buffer control chamber retained their globular appearance after 90 min (Figure. 6a). By contrast, PMNs activated with IL-8/CXCL8 developed an elongated cell shape with a contracted tail (Figure 6b). Blockade of PMNs with TFMK (Figure 6c) or pyrrophenone (Figure 6d) prevented the deformability of cell shape caused by IL-8/CXCL8.

IL-8/CXCL8 caused F-actin polymerization and translocation of cytosolic F-actin to the inner leaflet of PMN membrane (Figure 6). However, inhibition of gIVaPLA_2_, which prevented the elongation of PMN (see Figure 6c-d), did not block IL-8/CXCL8 -induced F-actin polymerization (Figure 6g-6h). Thus, while activation of gIVaPLA_2 _is essential for the change in shape of PMNs, F-actin polymerization, another essential step for cell migration, is not regulated by gIVaPLA_2_.

## Discussion

The objective of this study was to examine the functional role of gIVaPLA_2 _in the regulation of PMNs migration caused by IL-8/CXCL8. Prior studies have reported the signaling role of upstream kinases, ERK-1/2, p38 MAPK, and PI3K [1,5,6], in the initiation of cell migration; however, the downstream regulation of PMN migration elicited by IL-8/CXCL8 has not been elucidated previously.

We used a transwell-migration chamber [28] and determined whether inhibition of activated gIVaPLA_2 _by TFMK or pyrrophenone blocked PMN migration caused by IL-8/CXCL8. We also examined the functional role of gIVaPLA_2 _in causing in PMN elongation and F-actin polymerization, which both are necessary for PMN migration [34,35]. While the specific mechanism causing the PMN change in cell shape was not elucidated fully in these studies, we found that inhibition of gIVaPLA_2 _is sufficient to block change in cell shape caused by IL-8/CXCL8 even in the presence of F-actin polymerization (Figure 6).

Studies were designed using IL-8/CXCL8, a potent chemoattractant of PMNs. Prior studies have suggested that IL-8/CXCL8 is rather weak stimulator of human PMNs in comparison to rodent models [36]. Activation of PMNs with IL-8/CXCL8 did not elicit arachidonic acid or LTB_4 _secretion in human PMNs. Thus, our findings suggest that IL-8/CXCL8 caused transmigration of PMNs by a process that does not involve activation of arachidonate synthesis.

Cytosolic gIVaPLA_2 _is a critical messenger protein for cellular adhesion [21,22,27]. We have shown recently that neutrophil or eosinophil binding to ICAM-1 is mediated through activation of ERK-1/2 and subsequent phosphorylation of gIVaPLA_2 _[22-24,27]. In all prior cases, we have found that stimuli that upregulate cell adhesion CD11b expression also induce the activation of gIVaPLA_2 _[22,24,25,27]. However, the role of gIVaPLA_2 _to mediate PMN migration has not been previously reported.

Initial experiments were performed to confirm that upstream kinases, ERK-1/2, p38 MAPK, and PI3K, were activated by the concentration of IL-8/CXCL8 used in these studies (Figure 2). Immunoblotting analysis demonstrates that IL-8/CXCL8 elicited rapid phosphorylation of ERK-1/2, p38 MAPK, and Akt/PKB (Figure 2), confirming that these kinases were activated in these experiments; however, the downstream signaling pathway for cell migration has not been characterized. In this study, we used two-unrelated pharmacological inhibitors of gIVaPLA_2_, TFMK and pyrrophenone, to elucidate the role of gIVaPLA_2 _in cell migration. Transmigration of PMNs was blocked substantially in the presence of upstream phosphorylation of ERK-1/2, p38 MAPK and Akt/PKB (a target protein of PI3K) using inhibitors of activated gIVaPLA_2_. Accordingly, these data indicate a downstream regulatory role for gIVaPLA_2 _in *in vitro *PMN migration subsequent to activation of upstream kinases by IL-8/CXCL8.

Immunoblotting analysis demonstrated that IL-8/CXCL8 caused phosphorylation and translocation of cytosolic gIVaPLA_2 _to the nuclear component of PMNs [27]. It has been shown that gIVaPLA_2 _inhibition effectively blocked cell adhesion and secreted mediators after cell activation [21,24,31]. We have demonstrated that inhibition of gIVaPLA_2 _blocked both gIVaPLA_2 _enzymatic activity (Figure 4) and cell migration (Figure 5) elicited by IL-8/CXCL8 in concentration dependent manner. These data thus imply that activated gIVaPLA_2 _is an essential intermediate step in PMN migration in vitro.

Prior studies have demonstrated that interference with F-actin rearrangement could contribute to decrease cell migration [37]. We observed that inhibition of gIVaPLA_2 _with TFMK or pyrrophenone did not prevent the rearrangement of F-actin assembly elicited by IL-8/CXCL8. F-actin polymerization still was evident around the edges of inner cell membrane (Figure 6g-h). These findings suggest that while IL-8/CXCL8 caused change in cell shape, gIVaPLA_2 _does not directly regulate the rearrangement of the actin cytoskeleton in PMNs.

It is important to note some limitations to our *in vitro *models of PMN migration. We used transwell chamber *in vitro *to study transmigration of human PMNs. Migration *in vitro *occurred in the absence of β_2_-integrin ligation, which is the first step (adhesion) in cell migration *in vivo *[38]. *In vivo *conditions are a more complex environment, and it is not possible to extrapolate these data directly to the human situation. Studies *in vivo*, however, do not allow for stimulus isolation to specify mechanisms and sequence of cell migration. In these studies, maximal migration of PMNs from the upper chamber to the lower chamber containing IL-8/CXCL8 was ~50%. This is comparable to other chemoattractants, i.e., FMLP, C5a and LTB_4 _[39]. The initial number of cells (5 × 10^4 ^cells) was constant in all studies, and was sufficient to cover the area of a chamber (96-well chamber) for optimal PMN migration.

## Conclusion

Our data demonstrate that gIVaPLA_2 _activation caused by IL-8/CXCL8 (subsequent to activation of upstream kinases) may be an essential step in human PMN migration. Change in PMN cell shape and migration correspond to increased activity of cytosolic gIVaPLA_2 _and translocation of gIVaPLA_2 _to the nuclear membrane.

## Abbreviations

PMNs: polymorphonuclear leukocytes; gIVaPLA_2_: Group IVa Phospholipase A_2_; ERK-1/2: Extracellular Signal-Regulated Kinase; MAPK: Mitogen-Activated Protein Kinase; TFMK: Arachidonyl trifluoromethylketone; [^14^C]-PAPC: 1-palmitoyl-2- [1-14C]arachidonyl-phosphatidylcholine.

## Competing interests

The authors declare that they have no competing interests.

## Authors' contributions

NMM and ARL equally contributed to the concept and design of the study, and to the manuscript writing. NMM and AYM performed the data analysis. AYM and LNM performed the assays for gIVaPLA_2 _enzyme activity, PMN migration, F-actin polymerization, and change in cell shape. CO and DB performed the isolation of PMNs and western blot analysis. XZ and SD participated in the migration assay. All authors read and approved the final manuscript.

## Author's information

Angelo Y. Meliton, MD

Nilda M. Muñoz, MS

Lucille N. Meliton, MD

David Binder, BS

Christopher Osan, BS

Xiangdong Zhu, MD

Steven M. Dudek, MD

Alan R. Leff, MD
